# The culture of scientific research

**DOI:** 10.12688/f1000research.6163.1

**Published:** 2015-03-13

**Authors:** Catherine Joynson, Ottoline Leyser

**Affiliations:** 1 Nuffield Council on Bioethics, 28 Bedford Square, London, WC1B 3JS, UK; 2 Steering Group on the Culture of Scientific Research, Sainsbury Laboratory, Cambridge University, Bateman Street, Cambridge, CB2 1LR, UK

**Keywords:** Research, culture, science, integrity, misconduct, ethics, Nuffield Council on Bioethics

## Abstract

In 2014, the UK-based Nuffield Council on Bioethics carried out a series of engagement activities, including an online survey to which 970 people responded, and 15 discussion events at universities around the UK to explore the culture of research in the UK and its effect on ethical conduct in science and the quality of research. The findings of the project were published in December 2014 and the main points are summarised here. We found that scientists are motivated in their work to find out more about the world and to benefit society, and that they believe collaboration, multidisciplinarity, openness and creativity are important for the production of high quality science. However, in some cases, our findings suggest, the culture of research in higher education institutions does not support or encourage these goals or activities. For example, high levels of competition and perceptions about how scientists are assessed for jobs and funding are reportedly contributing to a loss of creativity in science, less collaboration and poor research practices. The project led to suggestions for action for funding bodies, research institutions, publishers and editors, professional bodies and individual researchers.

## Project origins

In December 2014, the Nuffield Council on Bioethics, an independent body that explores ethical issues in biology and medicine, published the findings of a series of engagement activities that explored the culture of scientific research in the UK. There were several motivations behind the project. First, two of the Council’s previous inquiries considered the factors that affect how and what scientific research is carried out, and identified a need for further examination of the issues. *Emerging biotechnologies: technology, choice and the public good* ^1^ looked at the factors affecting the direction of research, such as funding sources, interest in the economic and societal impact of research and public expectation, and found that these can lead to ‘overpromising’ by researchers that risks undermining public trust in science and misleading national policy. *Novel neurotechnologies: intervening in the brain* ^2^ looked at the pressures on academic scientists to demonstrate the practical and economic impacts of their work and the potential for these pressures to encourage premature emphasis upon commercial applications, or publication bias towards positive or newsworthy findings. Second, the Council was aware of a wider public debate about integrity and misconduct in research, sparked perhaps by several high profile cases of research misconduct, and felt it to be an area where the Council might usefully contribute. It commissioned a background paper on scientific research integrity to inform the Council’s deliberations ^3^.

Although the Council recognised that there were already a number of important initiatives that were promoting rigour and integrity within the research community ^4, 5^, it suspected that aspects of the *culture* in which research is funded, practised and communicated may be undermining efforts to raise the conduct of science to the highest ethical standards. After consulting organisations that work closely with the scientific community, it appeared that the Council’s concerns were shared by others, and that there was a lack of evidence about the experiences of individual researchers in terms of the pressures and challenges they faced in the course of their work. The Council decided to embark upon a series of engagement activities to explore the issues further.

## Project methods

In October 2013 the Council set up a Steering Group, which included members and staff of the Council, as well as staff of the Royal Society, Society of Biology, Institute of Physics, Royal Society of Chemistry and Academy of Medical Sciences, to design and oversee a project exploring the culture of research. The aim of the project was “to foster constructive debate among all those involved in scientific research about the culture of research in the UK and its effect on ethical conduct in science and the quality, value and accessibility of research; and to advance current debate through wide dissemination of the outcomes of these discussions”. ‘Science’ and ‘scientific research’ were not strictly defined in order to allow anyone involved in science or a related discipline to take part in the activities.

The activities of the project included:

- An online survey that was open from March to July 2014 and received 970 responses ( Supplementary file 1 lists the questions used in the online survey and an analysis of the online responses is available at: http://nuffieldbioethics.org/wp-content/uploads/The_culture_of_scientific_research_survey_analysis_for_web.pdf).
- Fifteen discussion events co-hosted with universities around the UK between June and September 2014, involving around 740 speakers and participants.
- Evidence-gathering meetings with funding bodies, publishers and editors of scientific research, and academics from the social sciences with expertise in the practice and culture of scientific research.

A report summarising the views expressed in the survey responses and by those who took part in the events and meetings was published on 4 December 2014 ^6^. The following documents were published on the same day ( www.nuffieldbioethics.org/research-culture):

- A detailed analysis of the survey responses carried out by the research consultancy Research By Design.
- A summary of the university discussion events written by the Nuffield Council on Bioethics Secretariat.
- A background paper covering recent research, policy developments and current debate relevant to the culture of scientific research in the UK, also written by the Nuffield Council on Bioethics Secretariat.

It should be noted that the people who took part in the survey and discussion events were self-selecting participants and therefore cannot be assumed to be representative of the wider researcher population. Most of those who took part in our activities were involved or interested in research being undertaken by higher education institutions (HEIs), which carry out around a quarter of UK research and development. A large proportion of the survey respondents and event participants work in either bioscience or medicine and are early-career researchers, which is reflective of the demographics of the HEI research community. Notwithstanding these limitations in the data, we believe some important themes and ideas emerged during the project that are relevant to many areas of academic research.

## Project findings

A full account of the project findings can be found on the Nuffield Council on Bioethics website. The key points are outlined below.

### What is high quality science?

In order to frame later questions about the effects of research culture on the production of high quality science, it was important to establish at the beginning of the survey what respondents regarded to be ‘high quality science’. When respondents were asked to select five words from a list that best describe their understanding of high quality research, the five most frequently selected words were:

1 Rigorous
2 Accurate
3 Original
4 Honest
5 Transparent

During the project activities it emerged that several other components are thought to be particularly important in the production of high quality science, namely: *collaboration, multidisciplinarity, openness* and *creativity*. For example, increased collaboration was the most common answer given when survey respondents were asked what feature of the UK research environment is having the most positive effect on science. The respondents (a quarter) who raise this think collaboration is leading to better communication between researchers, greater sharing of data and methodologies, less competition between different research teams, and reduced feelings of isolation among researchers.

Positive attitudes towards openness were expressed in answer to several of the survey questions. For example, 61 per cent of survey respondents thought that the move towards open access publishing is having a positive or very positive effect overall on scientists in terms of encouraging the production of high quality research. In addition, almost two thirds of respondents believe data sharing policies in the UK are having a positive or very positive effect overall. Respondents believe increased transparency and data sharing are facilitating the dissemination of results, enabling research to be accomplished more quickly and cost effectively, and allowing greater scrutiny of research findings. A greater proportion of respondents aged under 35 think data sharing policies are having a very positive effect overall than those aged over 45 (15 per cent vs. 7 per cent).

The description of high quality science by the participants of our project has notable similarities with the five themes that were identified during the UK Government’s consultation for its science and innovation strategy, also published in December 2014. The themes are excellence, collaboration, agility, place and openness ^7^.

### The motivations of scientists

The motivations of scientists provide additional insights into how they view research, and the majority of the survey respondents clearly chose a career in science in order to find out more about the world around them. When respondents were asked to rank phrases to describe what they believe motivates them in their work, the top three were:

1 Improving my knowledge and understanding
2 Making scientific discoveries for the benefit of society
3 Satisfying my curiosity

### Competition

High levels of competition in scientific research emerged as a strong theme running through all the project activities. Applying for funding is thought to be very competitive by the majority of the survey respondents (94 per cent), as is applying for jobs and promotions (77 per cent). Around nine in ten think making discoveries and gaining peer recognition is quite or very competitive.

High levels of competition for jobs and funding in scientific research are believed by survey respondents both to bring out the best in people and to create incentives for poor quality research practices, less collaboration, and headline chasing. For example, behaviours such as rushing to finish and publish research, employing less rigorous research methods and increased corner-cutting in research were raised by 29 per cent of survey respondents who commented on the effects of competition on scientists.

### Funding

When asked which features of the UK research environment are having the most negative effect, the most common answer given by survey respondents (31 per cent) was the lack of funding available. In addition, concerns were expressed about a loss of creativity and innovation in science caused by strategically-directed funding calls, short-term funding, and trends towards funding of safer research projects and established research centres. For example, when asked what they would like to change about the UK research environment, over 42 per cent of respondents comment on funding issues, with some expressing a desire for more funding for ‘riskier’ projects. There is a feeling that funding bodies have become more conservative and favour safer research projects, where results are almost guaranteed in advance, but this approach, respondents believe, can hamper scientific development.

When we tried to verify whether concerns about funding trends had a factual basis, we found that funding bodies offer a wide range of research grants, fellowships, studentships, training and other programmes, and support many different types of research. However, detailed information about trends in levels of funding and types of research being funded over time was not readily available from most funding bodies.

### Assessment of research

In a competitive system, the criteria used to assess the quality and value of science influences what science is pursued and how scientists behave. Peer review is the mainstay of most research assessment processes, including those carried out by journals to assess which work to publish, by funding bodies to determine which proposals to fund or how much core funding to allocate, and by institutions to decide who to appoint or promote to academic positions.

***Publish or perish.*** Throughout the project we heard repeatedly that publishing in high impact factor journals is still thought to be the most important element in determining whether researchers gain funding, jobs and promotions, along with article-level metrics such as citation numbers. This has created a strong pressure on scientists not only to ‘publish or perish’, but to publish in particular journals. Given that acceptance of a paper in a prominent journal typically requires that the findings represent a major, and possibly newsworthy, advance in knowledge, this trend is believed to be resulting in important research not being published, such as research with negative findings or research that replicates or refutes others’ work. Assessment processes that focus on publications in particular journals are also thought to be creating disincentives for multidisciplinary research, authorship issues and a lack of recognition for non-article research outputs.

***Peer review.*** Seventy-one per cent of the survey respondents believe the peer review system in the UK is having a positive or very positive effect overall on scientists in terms of encouraging the production of high quality science. However, concerns were raised about unconstructive reviewer comments and shortages of peer reviewers. Participants at several of the events raised the need for a review of the way in which peer review is carried out in the sciences. In particular, we heard support for both double-blind and open peer review as alternatives to the current system. The importance of peer reviewers being given training, time and recognition for their work was emphasised.

***The REF.*** In 2013, the UK higher education funding bodies undertook a process for assessing research quality, the Research Excellence Framework (REF), to inform the allocation of core funding to HEIs from 2015. The process involved peer review of each institution on the basis of 1) the outputs of research (such as journal publications, datasets and patents), 2) the impact of past research on the economy, society and culture, and 3) the vitality and sustainability of the research environment. The REF was a frequent topic of discussion during the project activities. We heard that the REF is thought to be a key driver of the pressure on researchers to publish in high impact journals, with many unaware or untrusting of the instructions given to REF panels not to make any use of journal impact factors in assessing the quality of research outputs. When asked for their views specifically on the REF, almost 40 per cent said they think the REF is having a negative or very negative effect overall on scientists in terms of encouraging the production of high quality science (with 25 per cent saying they believe it is having a positive or very positive effect). It was raised in several of the discussion events that the REF may be disadvantaging multidisciplinary work.

***Impact.*** The assessment by the REF of the impact of research beyond academia has been controversial, as has similar attempts by other funding bodies to consider the impact of research, such as the Research Councils’ ‘Pathways to impact’ section of grant applications. We found that the assessment of the societal and economic impact of research are welcomed by some, but others believe it is creating a culture of short-termism, pushing aside interest in curiosity-driven research, and resulting in researchers exaggerating the potential applications of research in grant proposals. The Research Councils we spoke to believe they have a duty to explain to the public and the Government the impact of public investment in science. They emphasised that this is done mostly retrospectively, and applicants are not expected to be able to predict at the application stage the economic or societal impacts that research will achieve. The results of the REF, published on 18 December 2014, found that the vast majority of submissions demonstrated impacts that were outstanding (4*) or very considerable (3*) ^8^. We await with interest the findings of research being undertaken by the Higher Education Funding Council for England (HEFCE) to evaluate the strengths and weaknesses of the REF process, as well as a review of the role of metrics in research assessment ^9^.

***Recognising other professional activities.*** Almost half of the survey respondents believe provision of professional education, training and supervision in the UK is having a positive or very positive effect overall on scientists in terms of encouraging the production of high quality science. However, although staff development, PhD awards and research collaboration are already recognised by the REF in the ‘Environment of research’ category and often feature in university promotion criteria, there was a clear perception among the event participants that this kind of activity is undervalued. It was suggested during the discussion events that research organisations should pay closer attention to and value the hard-to-measure and often invisible ways in which researchers contribute to the production of high quality science. This may include mentoring, training, teaching, peer review, university administration, public engagement and contributing to the work of national bodies and policy makers.

### Research integrity

There is a wide range of activities that could be considered to constitute research misconduct or poor quality research practice. This includes data fraud, poor experimental design, corner-cutting in research methods, inadequate replication of research, ‘cherry picking’ results, inappropriately slicing up data to create several papers, authorship issues, plagiarism, over-claiming the significance of work in grant proposals and papers, and carrying out poor quality peer review.

Research integrity came up frequently at the discussion events. Participants noted that honesty and trust is fundamental to science, and high profile cases of research misconduct may be undermining public trust in science. The view was expressed that high levels of competition for scarce resources put scientists under immense pressure which means that scientists are “bound to behave less well”.

This was confirmed by the survey findings. Fifty-eight per cent of respondents to the survey are aware of scientists feeling tempted or under pressure to compromise on research integrity and standards, although evidence was not collected on any behaviour associated with these findings. Twenty-six per cent of respondents have themselves felt tempted or under pressure to compromise on research integrity and standards. A higher proportion of respondents aged under 35 years (33 per cent) stated they had felt tempted or under pressure in comparison with those aged above 35 years (21 per cent). Thirty-eight percent of the survey respondents who comment on research integrity and standards think the ‘pressure to publish’ can encourage the fabrication of data, altering, omitting or manipulating data, or ‘cherry picking’ results to report. Thirty-one per cent of respondents think there is pressure to focus on and report positive results, rather than negative results, and that researchers rushing to publish results may not conduct appropriate replications and scrutiny of their work.

The first sector-wide research guidance for universities, *The Concordat to Support Research Integrity*, was published in 2012 ^10^. Sixty per cent of survey respondents think that initiatives that promote integrity in science in the UK, such as codes of conduct, are having a positive or very positive effect overall on scientists in terms of encouraging the production of high quality science. In addition, over half of survey respondents think ethical review processes in the UK are having a positive or very positive effect. This view is especially prevalent among respondents from social science, psychology, medicine and bioscience, and respondents note that ethical standards in the UK are thought to be high in comparison to other countries.

Suggestions for improving research integrity in the UK were made by event participants. Universities, they suggested, have a responsibility to create conditions to support ethical research conduct and demonstrate clearly the consequences of poor research practice. Training in good research practice was thought to be important in this regard, particularly for PhD students, but time pressures on senior scientists might be preventing this from happening at the moment. Universities might also be more open about how individual cases are resolved.

### Researcher careers

The number of academic staff across all disciplines employed in English HEIs has risen from around 105,000 in 2003–04 to around 126,000 in 2012–13 ^11^. Around 30 per cent of science PhD graduates go on to post-doctoral research positions, but only around four per cent of science PhD graduates proceed to permanent academic posts with a significant research component ^12^.

When asked if there is anything they would like to change about the UK research environment, more than a third of survey respondents cite issues related to career structure and progression. The following concerns were frequently mentioned by survey respondents and event participants:

- Short-term contracts and job insecurity for post-doctoral researchers
- Reliance on external funding for job retention, which drives the ‘pressure to publish’
- Pressure to progress but high competition for jobs and funding
- The need to keep relocating in order to take up the next position
- Limited opportunities for women in particular to have career breaks
- Heavy workloads and long hours
- High ‘drop out’ rates

Almost twice as many female survey respondents as male respondents raise issues related to career progression and the short-term culture within UK research when asked which features of the research environment are having the most negative effect on scientists. In addition, fifty-four per cent of respondents think the way scientists are assessed for promotion during their career is having a negative or very negative effect overall on scientists in terms of encouraging the production of high quality science, compared to 22 per cent who think it is having a positive or very positive effect.

In terms of how issues relating to careers and workloads affect the production of high quality science, survey respondents believe that they contribute to a culture of short-termism, high levels of stress, a lack of time to think and the loss of talented individuals from academia, which in turn results in a loss of creativity and innovation. Respondents also raise the possibility that high levels of competition for jobs may encourage poor quality research practices.

Suggestions for ways of addressing some of these concerns were raised during our discussions. For example, mentoring of early career scientists and the provision of appropriate career advice was suggested at several of the events as a possible way to help mitigate anxieties and help researchers be realistic about their prospects for a career in scientific research. Mentoring and advice may also help people to plan and develop their career paths at an earlier stage, and ensure they gain experience that will be transferrable to other sectors. PhD students are already encouraged by some funders to spend time in other sectors in order to expand their skills and experience, and the Royal Society recently published guidance on how doctoral students’ careers expectations can be best managed so that they, and their supervisors, understand the wide range of careers to which a PhD can lead, both inside and outside of scientific research ^13^. However, the funders we spoke to reported less progress in this area among post-doctoral researchers.

There are a number of other existing initiatives that aim to improve researcher careers. *The Concordat to Support the Career Development of Researchers* ^14^, for example, was highlighted during the project as a positive development in improving the way in which researchers are promoted and recruited. A recent report on progress with the implementation of the Concordat found that, although significant transformation has been achieved, further challenges remain, for example with regards to researchers taking more responsibility for their own professional and career development ^15^.

Participants at some of the events noted that the number of women in science has increased and that the introduction of formalised research assessment systems may have helped to tackle gender biases, which may have formerly influenced decisions about funding allocation and career progression. The Athena SWAN Charter, a national scheme that recognises good employment practice for women working in science, was mentioned at a number of events and is seen as having a positive influence on diversity in science. However, a majority (57 per cent) of those who responded to a call for evidence that formed part of HEFCE’s review of metrics in research assessment were negative about increasing the use of metrics, and common among their concerns was that a further use of metrics could disadvantage under-represented groups: early-career researchers, women, those with disabilities, and black and minority ethnic (BME) academics ^16^. A workshop on equality and diversity organised by HEFCE as part of the review highlighted problems associated with ‘implicit bias’ and the possible unintended consequences of research assessment regimes ^17^. These concerns emphasise the importance of diversity in assessment methods.

## Observations and suggestions for action

Scientists told us they are driven by the desire to improve their knowledge and understanding, to make discoveries for the benefit of society and to satisfy their curiosity. High quality research was described as: rigorous, accurate, original, honest and transparent; and collaboration, multidisciplinarity, openness and creativity are thought to be important for the production of high quality science. Within this context, the findings of the project led us to make some general observations:

- In some cases the culture of scientific research does not support or encourage scientists’ goals and the activities that they believe to be important for the production of high quality science.
- A highly competitive environment with a narrow range of assessment methods are thought to be the main risk factors.
- There seem to be widespread misperceptions or mistrust among scientists about the policies of those responsible for the assessment of research.
- Among all the relevant stakeholders, concerns about the culture of research are often attributed to matters that they think are outside their control or are someone else’s responsibility.

**Figure 1.**
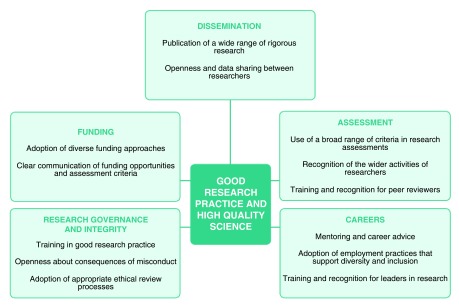
Suggestions for action to support good research practice and the production of high quality science.

We believe there is a collective obligation for the actors in the system to do everything they can to ensure the culture of research supports good research practice and the production of high quality science. As such, we provide a number of suggestions for action for funding bodies, research institutions, publishers and editors, professional bodies and individual researchers (see Figure 1). Key examples are:

- Funders: ensure funding strategies, policies and opportunities, and information about past funding decisions, are communicated clearly to institutions and researchers; and provide training for peer reviewers to ensure they are aware of and follow assessment policies.
- Research institutions: cultivate an environment in which ethics is seen as a positive and integral part of research; ensure that the track record of researchers is assessed broadly; and provide mentoring and career advice to researchers throughout their careers.
- Publishers and editors: consider ways of ensuring that the findings of a wider range of research meeting standards of rigour can be published; consider ways of improving the peer review system; and consider further the role of publishers in tackling ethical issues in publishing and in promoting openness among scientists.
- Researchers: actively contribute to the adoption of relevant codes of ethical conduct and standards for high quality research; use a broad range of criteria when assessing the track record of fellow researchers; and engage with funders, publishers and learned societies to maintain a two-way dialogue and contribute to policy-making.
- Learned societies and professional bodies: promote widely the importance of ensuring the culture of research supports good research practice and the production of high quality science; and take account of the findings of this report in relation to guidelines for members on ethical conduct and professionalism.

## Reaction so far

The Steering Group hopes that the findings of this project provide useful evidence that will advance future debate on the culture of scientific research in HEIs. At this early stage, the findings appear to have been received very positively.

The Presidents of five major science organisations (the Nuffield Council on Bioethics, the Royal Society, Society of Biology, Royal Society of Chemistry and Academy of Medical Sciences) jointly wrote a Foreword for the report of the project ^18^. In the Foreword, the Presidents welcomed the report, recognised the integrated view that the culture-wide approach of this project provides, and committed to consider the report’s suggestions for action in the context of their own communities.

To raise awareness of the findings among policymakers, a launch event was held in the Houses of Parliament on 4 December 2014. The event was hosted by Andrew Miller MP, Chair of the House of Commons Science and Technology Committee and around 45 invited individuals attended, including representatives of funding bodies, journals and publishers, professional bodies and learned societies, universities, select committees, and others such as government departments and NGOs. Participants noted the many positive aspects of the project findings, for example that researchers clearly care about doing good research and the challenges they face. In addition, many positive developments were also highlighted, such as trends in publishing towards open peer review and the publication of a larger range of research outputs. All agreed that it was a complicated area and a collective and co-ordinated effort to tackle the issues was required. Throughout 2015, the Nuffield Council on Bioethics, working in particular with the organisations represented on the project steering group, will be encouraging all relevant stakeholders to do just that. If you are interested in getting involved, please contact Catherine Joynson on cjoynson@nuffieldbioethics.org.
