# Supplementary material for: The culture of scientific research
Source: F1000Res. 2015 Mar 13;4:66. [Version 1] doi: 10.12688/f1000research.6163.1 (PMC4376168; doi:10.12688/f1000research.6163.1)
Supplement: Peer review history

# Peer review: Referee response for version 1

**DOI:** 10.5256/f1000research.6607.r7957

**Published:** 2015-03-26

**Authors:** Kate Bullen

**Affiliations:** 1 University Director of Ethics and Equality, Aberystwyth University, Aberystwyth, UK

This article reports the rationale and findings of a major piece of work undertaken by the Nuffield Council on Bioethics in 2014. The changes to the funding of research in the UK, due to recessionary pressures, and the increased emphasis on "research with impact" by major funders and the REF2014 process, provides the context for the work. The importance of the article lies in the way in which it provides a context and narrative to these wider changes. Government and funders develop and impose strategic policy decisions in a 'top-down' process; this article reports on how those decisions are experienced from the 'bottom-up'. 

The article presents a range of fundings derived from an extensive and comprehensive data collection process. The authors identify the limitations of the data set (self-selected participants for example), but nonetheless the study provides a rich source of stimulus material for discussion and debate. Some of the key findings include:

1. The age differential in assessments of the worth of data sharing (15% of <35 year olds positive compared to 7% of >45 year olds).
2. The importance of social benefit and the collective good as fundamental to scientific research.
3. The tension between achieving collective good whilst promoting and supporting individual career progression.
4. The dual role of competition as a spur to delivery of 'best work' versus the pressure to take short cuts to achieve outcomes.

These findings, in conjunction with the others reported, are clearly suggestive of a body of researchers who increasingly aware of the pressure to perform to an ever higher level. The article reports the tension experienced by researchers who are striving to generate work of value, but are also aware of the temptations of "cutting corners" to achieve. For this reason the article acts as a catalyst for further debate, and generates more questions than it answers. What are the unintended consequences of increasingly stringent research ethics review processes? What is the real impact of the 'impact agenda' on research? Do REF type exercises discriminate between researchers and their work in positive or negative ways? How effective is peer review, and is it crumbling under the weight of research being generated? These questions, together with the important issue of gender equality being raised through the ECU Athena SWAN award and the emphasis on gender in Horizon 2020, and the changing nature of higher education in the UK, are all importantly raised by this article. 

The authors end with a series of sensible suggestions for future development. The value of the article lies in the fact that these suggestions are made on the basis of evidence generated from the scientific community. How these suggestions, and variations of them, are adopted and assessed will be an important, and as yet undetermined, outcome of this report.

I have read this submission. I believe that I have an appropriate level of expertise to confirm that it is of an acceptable scientific standard.

# Peer review: Referee response for version 1

**DOI:** 10.5256/f1000research.6607.r7956

**Published:** 2015-03-23

**Authors:** Cornelia Lawson

**Affiliations:** 1 School of Sociology and Social Policy, University of Nottingham, Nottingham, UK

The report provides a summary of the findings of the Nuffield Council on Bioethics’ “The culture of scientific research” project. The report provides insights into the conditions faced by scientists in the UK and makes a number of constructive suggestions at all levels of the academic enterprise that could help improve the culture of research and ensure continued high quality research. The work done by the Council has already been of great importance for the discussion of scientific culture in the UK.

I have some issues with the structure and focus of the report that prevent me from accepting without reservations. First, the report summarises the items that are considered particularly important for high quality science: collaboration, multidisciplinarity, openness and creativity. The discussion throughout the report and especially the concluding chapters would benefit from referring back to these four elements and how they can be fostered. For example, it is not clear how the suggestions for action will improve the conditions for multidisciplinary research.

Second, the subsections in ‘project findings’ are not clearly divided. While the first two subsections look at what research is and why people do it, the remainder is dedicated to the various elements that have a negative effect on the culture of research and that need to be improved. I would therefore suggest to split the section into two. The competition subsection could then serve as an introduction to the other elements (funding, assessment, careers etc.) as it is defined by them. Competition is seen as bad due to the problems with funding, assessment and careers and not because it is bad *per se*. In fact the report finds that half the survey respondents see competition as a good thing, because it is a driver for high quality research. A clearer split of the section and a better discussion of the role of competition would therefore benefit the report as a whole. Finally, the box headings in Figure 1 should correspond to the sub-section headings in ‘project findings’ (which they partially already do).

Finally, I have one very specific comment regarding the funding section. At the end of the section it says that information on trends in levels of funding and themes supported was not available. However, considering that most of the survey respondents come from the biosciences a look at BBSRC funding data, available on their website, can give a first indication of a time trend. There it becomes clear that the number of funded project did not increase over time (unlike the number of academic staff) which may result in more funding competition.

I have read this submission. I believe that I have an appropriate level of expertise to confirm that it is of an acceptable scientific standard, however I have significant reservations, as outlined above.

# Peer review: Referee response for version 1

**DOI:** 10.5256/f1000research.6607.r7955

**Published:** 2015-03-19

**Authors:** Curt Rice

**Affiliations:** 1 Department of Languages and Linguistics, University of Tromsø, Tromsø, Norway

The opinion article *The culture of scientific research* contributes to a debate focused on the tension between doing research and holding researchers and research institutions accountable. I find that the piece does not particularly surprise me, but it's contribution is nonetheless important as it offers a somewhat less anecdotal treatment of the issues, as well as offering constructive suggestions about how to move forward.

The article reports on the results of a survey done among scientists in the UK, with 970 responses, and on the results of a series of 15 "discussion events" having around 740 speakers and participants.

Both the survey and the focus groups considered the nature of scientific careers and the circumstances in which scientists currently work and related these things not only to each other but to the nature and quality of research being carried out.

It is heartening to learn that scientists report their primary motivations as being the quest for knowledge and the application of that knowledge in the service of society. We want to understand stuff. That's what we do. And counting pages published and impact factors is a distraction. But it's more than that.

Some of the results of the study reveal the belief that our current infrastructure for funding moves us away from riskier curiosity-driven projects, towards more conservative research.

The perniciousness of impact factor is seen as pressuring scientists to try for particular journals, as well as leading researchers away from reproducibility projects or the examination of negative results.

The structure of careers -- especially with strings of post-docs and temporary positions -- yields a culture of short-termism. I think this is a very serious point. If early career scientists have to apply for renewal or new positions every 2-3 years, they have to get results within the first year of a position to have something published by the time they apply for their next job. This is not good for the quest for knowledge and understanding, and I think this point is under-appreciated in the debate about the nature of an academic career. 

Another important topic the the article addresses is the pressure to take ethical short cuts. This in turn is one of the core issues in the authors' suggestions for improving our system. They stress the importance of training in ethical norms and practices and advocate for explicit engagement with this aspect of researcher training and practice.

In addition to encouraging us to nurture ethical training and practice, the authors also would like to see more training to do peer review and broader evaluation in connection with hiring, advancement and the awarding of grants.

The nature of research is being manipulated and damaged by the nature of the context in which research is carried out. This article investigates that claim more thoroughly, provides evidence, and offers constructive suggestions about what could be different.

Read this piece. And talk about it with your research communities.

I have read this submission. I believe that I have an appropriate level of expertise to confirm that it is of an acceptable scientific standard.
